# Supplementary material for: Multi-channel intraneural vagus nerve recordings with a novel high-density carbon fiber microelectrode array
Source: Sci Rep. 2020 Sep 23;10:15501. doi: 10.1038/s41598-020-72512-7 (PMC7511947; doi:10.1038/s41598-020-72512-7)
Supplement: Supplementary file 1 — Supplementary file [file 41598_2020_72512_MOESM1_ESM.pdf]

SUPPLEMENTARY FIGURES

Title:
Multi-channel intraneural vagus nerve recordings with a novel high-density carbon fiber microelectrode array

Authors:
Ahmad A. Jiman[1,2,3], David C. Ratze[2,4], Elissa J. Welle[1,2], Paras R. Patel[1,2], Julianna M. Richie[1,2], Elizabeth C. Bottorff[1,2], John P. Seymour[1,4,5], Cynthia A. Chestek[1,2,4], Tim M. Bruns[1,2,*]

1- Department of Biomedical Engineering, University of Michigan, Ann Arbor, MI, USA
2- Biointerfaces Institute, University of Michigan, Ann Arbor, MI, USA
3- Department of Electrical and Computer Engineering, King Abdulaziz University, Jeddah, Saudi Arabia
4- Department of Electrical Engineering and Computer Science, University of Michigan, Ann Arbor, MI, USA
5- Department of Neurosurgery, University of Texas Health Science Center, Houston, TX, USA

[*]Corresponding Author:
Tim M. Bruns, Ph.D.
NCRC - B10 - A-169
2800 Plymouth Road
Ann Arbor, MI 48109, USA
Phone: (734) 647-8727
Email: bruns@umich.edu

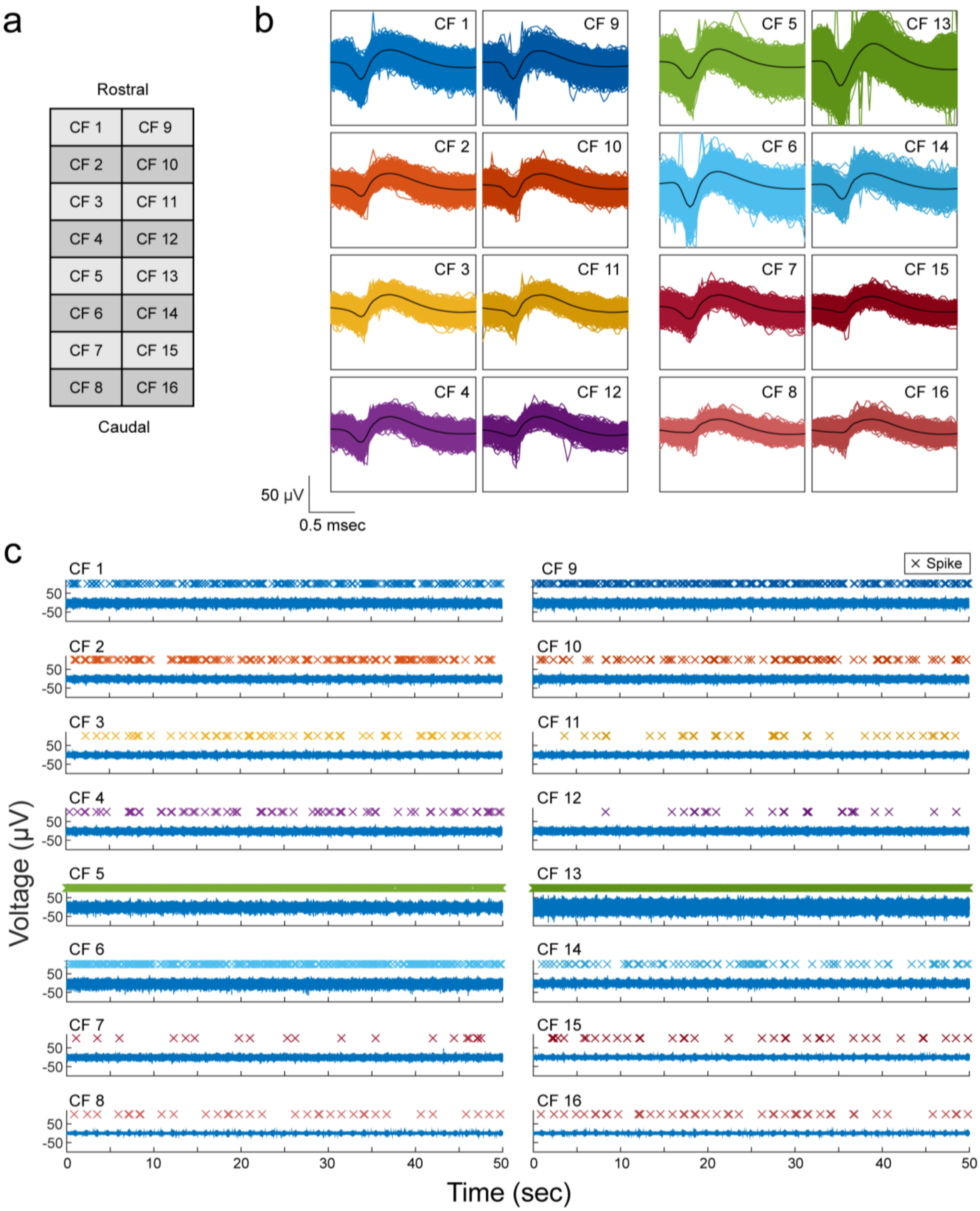

Supplementary Figure S1. Carbon Fiber Microelectrode Array (CFMA) with neural activity on all recording carbon fibers (CFs). (a) The CF layout in a CFMA. **(b)** Sorted neural cluster on each CF. **(c)** Segment of filtered signal on each CF. The firing of spikes are unique across CFs.

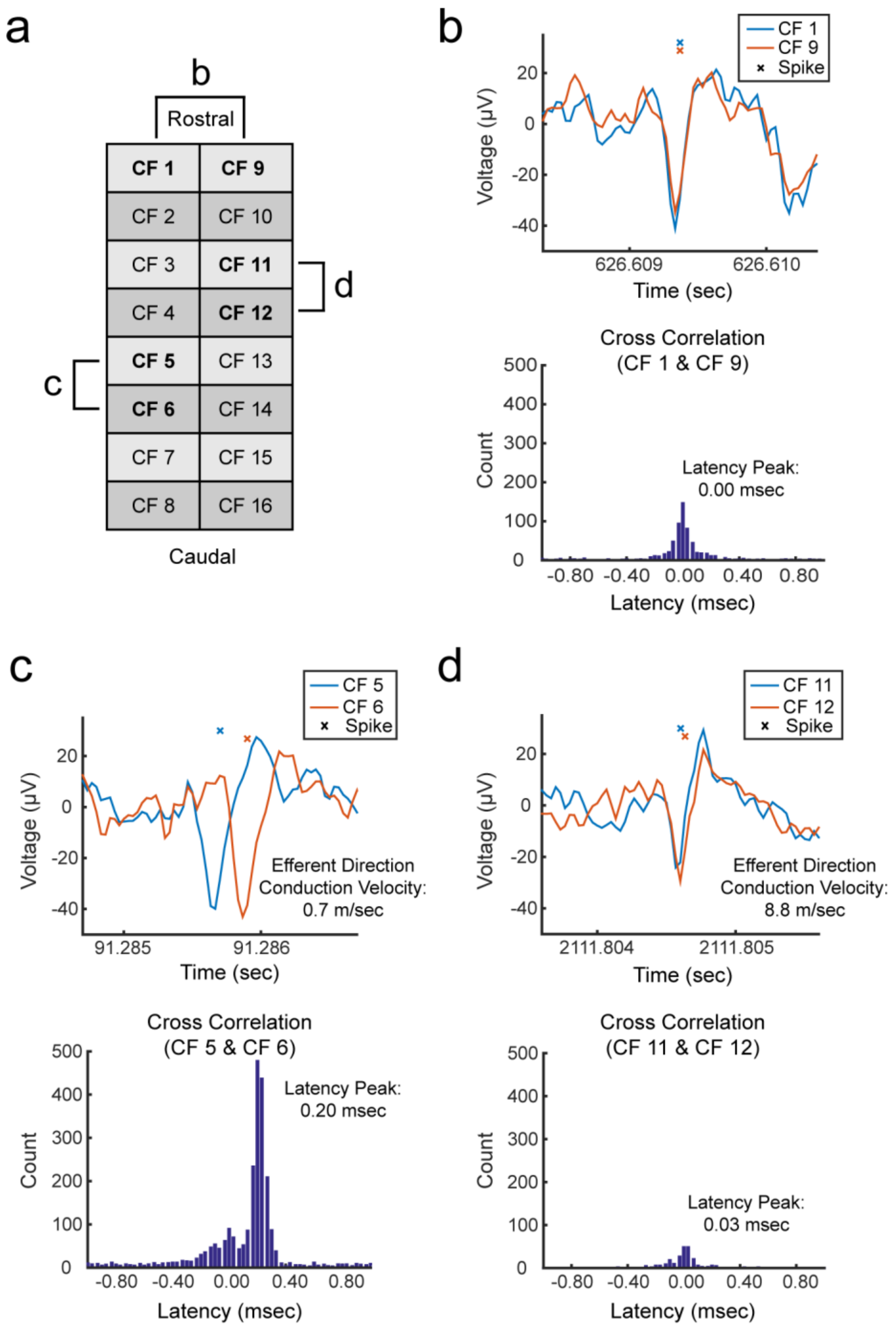

Supplementary Figure S2. Neural recording on opposite rows of a CFMA. (a) The 2-row carbon fiber (CF) configuration in a CFMA. **(b)** Two CFs on opposite rows showing coinciding spikes with different amplitudes, suggesting that these spikes are generated from a neuron located between these opposite carbon fibers. **(c)** Adjacent carbon fibers showing propagating spikes in the efferent direction at a conduction velocity of 0.7 m/sec along one row of a CFMA. (d) Propagating spikes on the other row with a conduction velocity of 8.8 m/sec in the efferent direction. The propagating spikes in c and d are independent of each other.

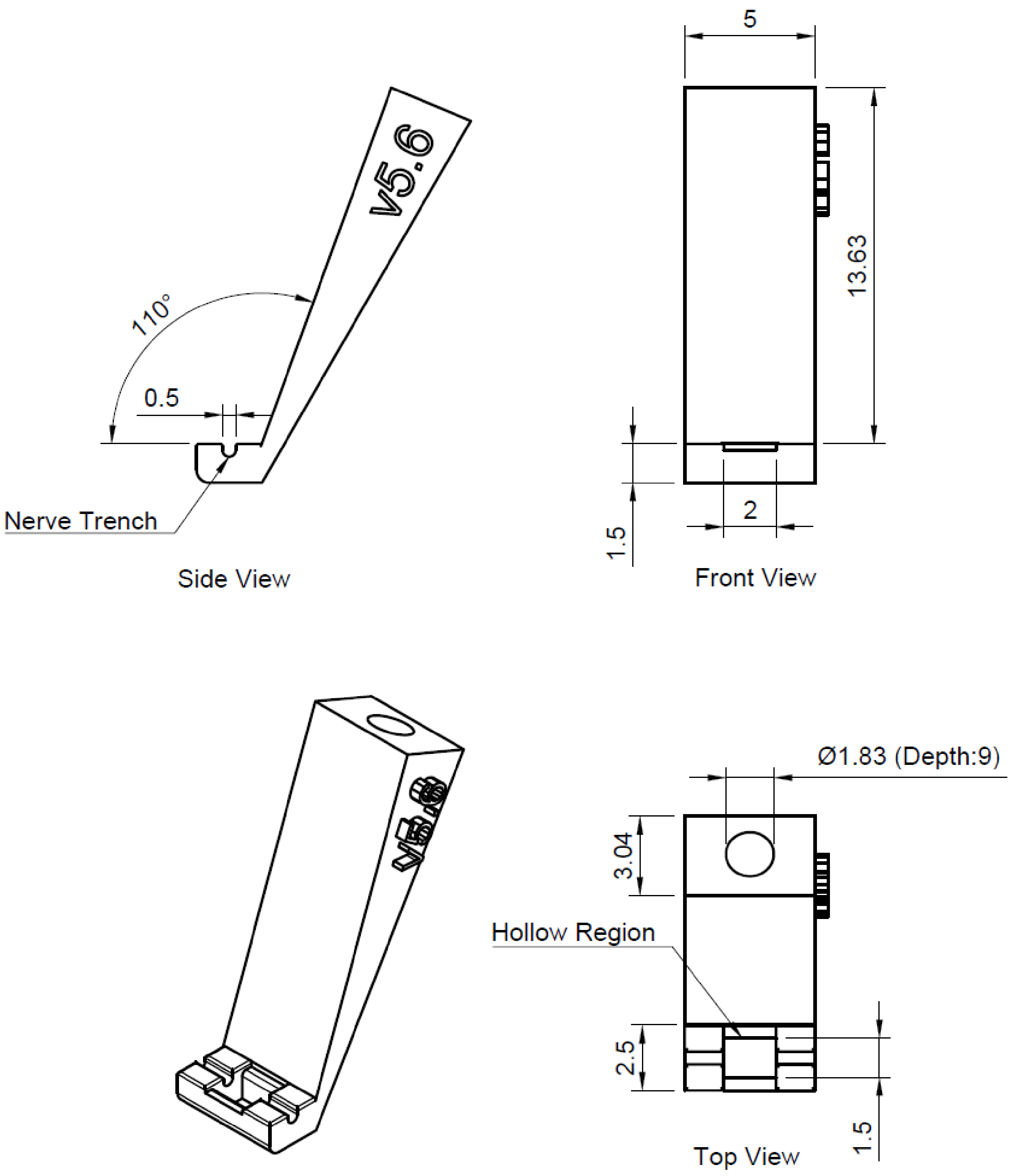

Supplementary Figure S3. Nerve-holder design for inserting CFMA in a rat cervical vagus nerve. Dimensions are in millimeters (mm).
